# Nitro-Glycerin in Bright’s Disease

**Published:** 1887-01

**Authors:** 


### *NITRO-GLYCERIN IN BRIGHT'S DISEASE.*

The employment of nitro-glycerin in chronic renal disease, for the valuable service in dispelling or moderating uræmic symptoms, is based upon the fact that high arterial tension is the constant concomitant of uræmia. How far it is desirable to habitually employ a remedy having so pronounced an action upon the circulation as nitro-glycerin, is yet undetermined; but experience of its value in the diseases which are marked by abnormal arterial tension is accumulating. The latest contribution is by Dr. Kinnicutt, of New York, who has studied the effects of the drug in several cases, with results which harmonize with those obtained by Rossbach and Burzbinski, to which allusion is made. The continued employment of the drug in slightly increasing quantity does not only relieve headache, dyspnœa, palpitation, and other symptoms referable to the uræmic state, but is marked by an increase in the diurnal excretion of urine, together with a notable diminution in the amount of albumen in it. Cases are given where the albumen was estimated quantitatively, and they show that in some the drug has a marked effect in its reduction. At the same time, as one shows, there is often great variability in the albuminuria of chronic nephritis, which renders it important that similar observations should be made on a large scale before trustworthy conclusions can be arrived at. The amount of nitro-glycerin administered should be just within the limit of producing any subjective symptoms. Dr. Kinnicutt's conclusions may be given in his own words: "1. That in nitro-glycerin, given in small doses and frequently repeated, we possess a powerful agent for lowering the increased blood-pressure which is very constantly associated with the development of uræmic symptoms. 2. That it has the power to control or relieve many of the paroxysmal disturbances of the nervous system which are included under the general term of uræmia; of these, headache and asthma are especially benefited by its use, the relief being more marked and continuous than that obtained either by opium or chloral. 3. That its influence upon the daily excretion of urine and serum-albumen in parenchymatous and interstitial nephritis is apparently to increase the former and diminish the latter. 4. That in the systematic and prolonged use of nitro-glycerin in appropriate doses, in chronic nephritis, we possess a means of maintaining more or less continuously a lowered blood-pressure, of often averting or relieving critical conditions, and there by prolonging life."—*Lancet*, June 12, 1886.

